# Gastric myeloid sarcoma mimicking pseudoachalasia in non-leukemic context: a singular case report

**DOI:** 10.1097/MS9.0000000000001830

**Published:** 2024-02-20

**Authors:** Nidal Jebrini, Husein Sarahneh, Mohanad Jaber, Motaz Natsheh, Ahmad Abu Ayyash, Sami Bannoura, Raghad Razem

**Affiliations:** a Faculty of Medicine, Palestine Polytechnic University, Hebron, Palestine; b Al-Ahli Hospital, Doha, Qatar; c Forensic Pathology

**Keywords:** AML, gastric myeloid sarcoma, psudoachalasia

## Abstract

**Introduction and importance:**

Myeloid sarcoma (MS) is a rare tumour associated with acute myeloid leukaemia (AML) and occasionally occurs independently. It typically affects skin, bone, lymph nodes, and rarely the gastrointestinal tract, with gastric cases being extremely rare. Notably, no reported instances associate pseudoachalasia with gastric myeloid sarcoma.

**Case presentation:**

A 20-year-old male presented with severe dysphagia, refractory vomiting, and weight loss. Diagnosed with type III achalasia via oesophageal tests, subsequent gastroscopy revealed a large gastric mass, later identified as gastric myeloid sarcoma through histopathology.

**Clinical discussion:**

MS, characterized by immature blast cells, poses diagnostic challenges without typical leukaemia symptoms. Diagnosis involves immunohistochemistry, employing markers like CD33, CD34, and CD43. Optimal treatments, such as chemotherapy or stem cell transplantation, aim to delay leukaemia progression. Gastric primary de-novo myeloid sarcoma is exceedingly rare, emphasizing the need for tailored treatment strategies.

**Conclusion:**

Gastric myeloid sarcoma is an exceptionally rare tumour, especially without concurrent acute myeloid leukaemia (AML), complicating its diagnosis. This case represents the first globally documented instance of gastric myeloid sarcoma causing pseudoachalasia. Documenting this unique clinical presentation is crucial for a better grasp of gastric myeloid sarcoma’s diverse manifestations.

## Introduction

Highlights

- Gastric myeloid sarcoma represents an exceedingly rare form of extramedullary neoplasm.
- Gastric myloid sarcoma commonly emerges as the initial manifestation of acute myeloid leukaemia (AML), occurrences in leukaemic patients are infrequent.
- Remarkably, this case stands as the inaugural global instance demonstrating the presentation of gastric myeloid sarcoma accompanied by pseudoachalasia.

Myeloid sarcoma (MS) represents a rare extramedullary solid tumour primarily constituted of immature myeloid cells. It is commonly associated with acute myeloid leukaemia (AML), occurring concurrently in a subset of AML cases (2–9%). Nevertheless, a small proportion of cases manifest as *de novo* or non-leukaemic presentations^1^. MS predominantly emerges in sites such as the skin, bone, lymph nodes, and rarely within the gastrointestinal tract, constituting only 2–8% of the total MS occurrences. Reports of gastric MS specifically are infrequent^2^. While the age range for AML diagnosis is broad, MS tends to have a relatively higher incidence among paediatric cases (up to 30%) compared to adults (2–5%)^3^.

The diagnosis of myeloid sarcoma heavily relies on histopathological and immunohistochemical analyses. In cases associated with leukaemia, the diagnosis is typically straightforward. However, isolated occurrences of MS pose diagnostic challenges, particularly considering the diverse anatomical sites in which this neoplasm can develop, alongside varying clinical presentations^4^.

Presented here is an exceptional case involving a 20-year-old male diagnosed with myeloid sarcoma situated in the gastric fundus? This rare presentation led to the obstruction of the lower oesophageal sphincter, resulting in type 3 achalasia (pseudoachalasia). Notably, this case lacked concurrent acute myeloid leukaemia, rendering the diagnosis notably challenging in this clinical context.

## Case presentation

**Figure 1:**
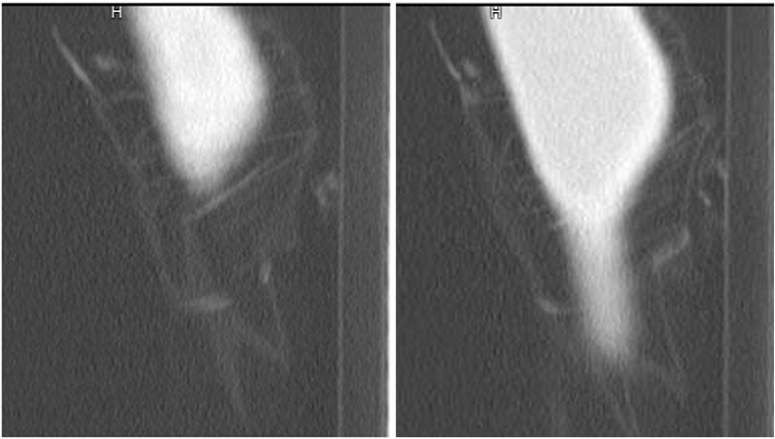
Bird peak appearance characteristic for pseudoachalasia on computed tomography scan.

A 20-year-old male with an unremarkable medical and surgical history was referred to our institution due to a protracted three-month history of escalating dysphagia to solids and liquids, accompanied by severe, refractory vomiting leading to a substantial 25% reduction in body weight. Complete blood count, liver and renal function reports of the patient were all within the normal range Preceding his referral, a barium swallow study exhibited findings suggestive of achalasia, displaying characteristic features of a dilated oesophagus and a distinct “bird’s beak” appearance at the GE junction (Figure 1).

To further explore the underlying pathology, the patient underwent sequential oesophageal manometry and gastroscopy as a day case procedure. OEsophageal manometry, conducted initially to avoid any potential negative impact of sedation on swallowing dynamics during endoscopy, revealed a type III achalasia pattern. This pattern, while consistent with primary achalasia, also bears resemblance to pseudoachalasia resulting from tumours located at the gastric cardia.

**Figure 2:**
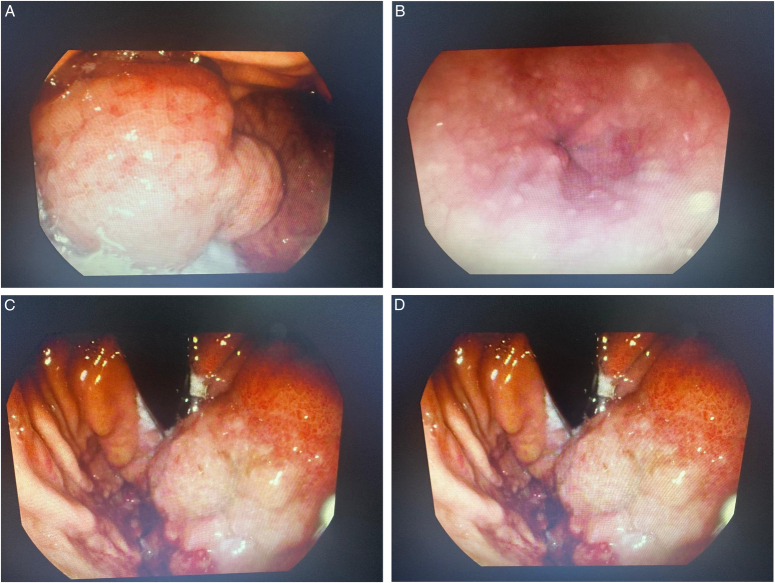
Massive mass obliterating gastric fundus (A), compressed GE junction by gastric mass (B),mass extension along lesser curvature into proximal gastric body (C, D).

Subsequent gastroscopy on the same day aimed to identify potential secondary causes. Notably, the examination revealed a dilated oesophagus with moderate resistance at the GE junction, hindering access to the stomach. Upon entry into the stomach, a conspicuous large rounded bulging mass was observed, obscuring the gastric cardia and fundus while exerting compression on the GE junction. This mass extended proximally along the gastric corpus, accompanied by another albeit smaller, yet substantial mass located distinctly at the greater curvature. Multiple biopsies were obtained from these lesions for further analysis and definitive diagnosis (Figure 2, illustrating the visual representation of the observed mass during the gastroscopy examination).

**Figure 3:**
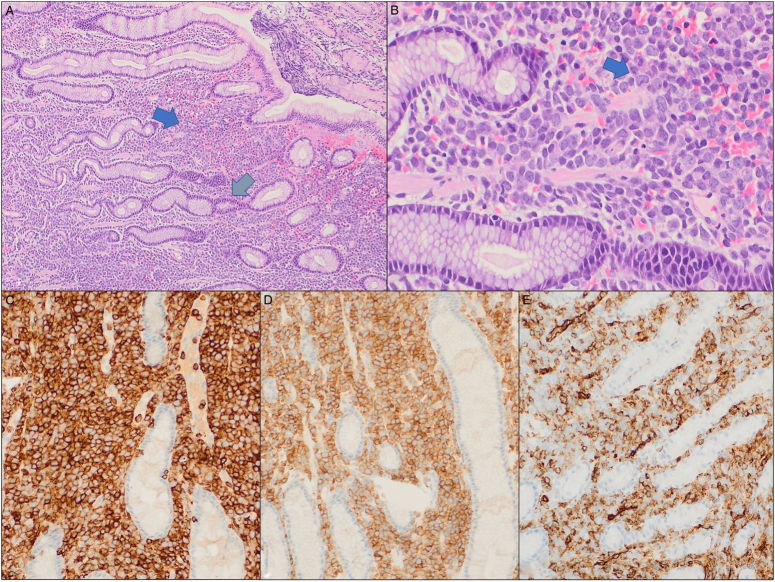
Myeloid sarcoma, stomach; (A) Sections show a cellular proliferation (blue arrow) in the lamina propria between gastric pits and glands (grey arrow) with no evidence of mucosal damage [hematoxylin and eosin (H&E), 10×]. (B) The malignant cells are medium to large with minimal amounts of cytoplasm, irregular nuclear contours with visible nucleoli. (H&E, 40×). (C–E) The atypical cells are positive for CD45, CD33, and CD34 immunostains supporting the diagnosis (20×). They were negative for CD3, CD20, CD79a, and TdT immunostains.

The comprehensive histopathological assessment, encompassing both morphological and immunohistological analyses of the obtained tissue biopsies, identified the presence of large atypical cells characterized by limited cytoplasm and irregular nuclear contours with discernible nucleoli. These cells exhibited notable immunoreactivity for CD33, CD34, and CD43 markers while demonstrating negativity for CD3, CD20, CD79a, and TdT immunostains. These distinct immunophenotypic features were consistent with the established diagnostic criteria indicative of gastric myeloid sarcoma. (Figure 3 illustrating the histopathological slides highlighting these specific and defining histological characteristics).

**Figure 4:**
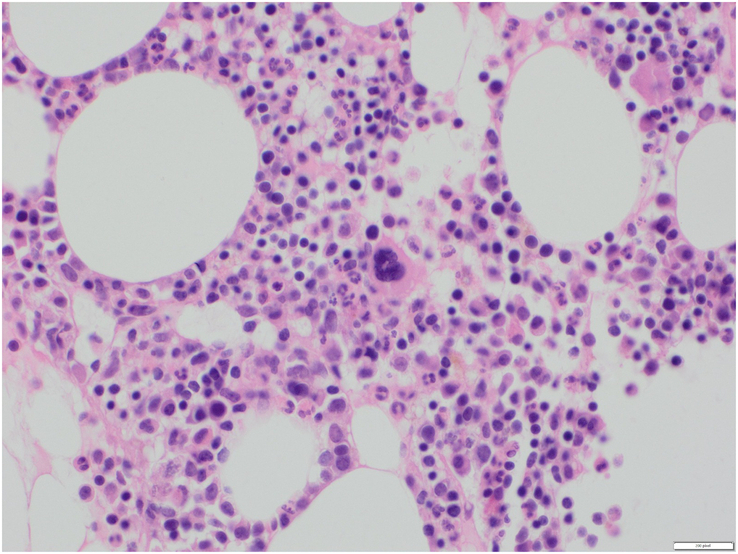
(Bone marrow 40×):Normocellular bone marrow with mature trlineage hematopoiesis. No infiltrate seen.

Since MS can present as initial presentation for AML, patient had bone marrow biopsy which revealed a negative result for AML (Figure 4). Due to the paucity of information in the existing literature regarding the optimal management approach for patients with this condition, the proposed plan entails the administration of a (7+3) regimen of chemotherapy (Idarubicin and Cytarabine). Notably, this approach does not involve gastric surgery.

In the course of the postoperative follow-up, the patient underwent a positron emission tomography (PET) scan, which revealed a negative metabolic uptake. This outcome is indicative of the success of the therapeutic intervention. Furthermore, given the intricate nature of the case, it is strongly advised to seek consultation from a specialized referral centre specializing in hematopoietic stem cell transplantation. This consultation is intended to comprehensively discuss and evaluate the potential necessity for a bone marrow transplant (even with negative bone marow biobsy) within the overarching management plan for the patient.

## Discussion

MS are characterized by the proliferation of immature blast cells originating from myeloid lineages, resulting in the replacement of the normal tissue architecture. These neoplastic lesions can occur at almost any anatomical site, with a predilection for diverse tissues. An analysis of 92 cases by Pileri *et al* ^1^. revealed common sites for MS, including the skin (28.2%), lymph nodes (16.3%), testes (6.5%), intestines (6.5%), bones (3.25%), and the central nervous system (3.25%). MS can affect individuals across a wide age range, with a notably higher incidence in paediatric patients, constituting up to 30% of cases, in contrast to the lower incidence of 2–5% observed in adults^5^. Typically, MS presents either concurrently with AML, chronic myeloid leukaemia, myeloproliferative neoplasms, or myelodysplastic syndrome (MDS) at the time of their initial diagnosis or during relapse^6^.

In this particular case, the patient initially presented with progressively worsening dysphagia, encompassing both solid and liquid foods, which eventually escalated to severe and refractory vomiting, accompanied by a substantial weight loss. Notably, the patient did not exhibit classic symptoms of leukaemia, such as fever, anaemia, or enlarged lymph nodes, and no evidence of bone marrow involvement was found. This atypical clinical presentation posed a significant diagnostic challenge, necessitating a comprehensive approach for accurate identification.

The utility of immunohistochemical markers played a pivotal role in establishing the diagnosis. Several markers were employed to discern the nature of the neoplastic cells. Large atypical cells demonstrated positivity for CD33, CD34, and CD43, which are widely recognized in the literature as crucial markers for diagnosing myeloid sarcoma^3^. Furthermore, these cells exhibited positivity for CD45, CD4, and variable reactivity for CD68, aligning with findings observed in the study conducted by Menasce *et al.* ^7^. Importantly, these cells tested negative for CD3, CD5, CD8, CD10, Pax5, MUM1, thereby excluding the possibility of B-cell and T-cell lineages. Additionally, the absence of CD30, further ruled out potential diagnoses related to Hodgkin’s lymphoma. This comprehensive immunohistochemical profiling was instrumental in confirming the diagnosis of myeloid sarcoma and differentiating it from other malignancies that may share overlapping clinical features.

The prognosis of MS within the context of AML has been a topic of interest, although its precise impact remains uncertain due to limited data. One retrospective study suggested that isolated MS may confer a relatively better prognosis compared to leukaemic AML, with nonsignificantly increased two-year event-free and overall survival rates^8^. However, the optimal treatment strategy for isolated MS remains elusive, given its rarity and varied clinical presentations. Another retrospective analysis indicated that systemic chemotherapy may prolong the time to progression to leukaemic AML, underlining its potential role in delaying disease evolution^9^. Early initiation of anti-leukaemic chemotherapy was associated with a lower likelihood of developing leukaemic AML and improved survival. Induction chemotherapy akin to that for leukaemic AML is now considered standard treatment for isolated MS, despite its propensity to progress to frank leukaemia^10,11^. Subclinical bone marrow involvement, detected in some cases, raises questions about the contribution of local therapies to disease progression^12,13^. Surgical and radiation therapies are accepted modalities for MS, though their precise roles in treatment algorithms remain unclear. They may offer symptomatic relief, initial debulking, or serve as salvage therapies in certain cases^14^. Allogeneic stem cell transplantation (Allo-SCT) is a viable consideration for both patients with leukaemia and those with isolated myeloid sarcoma, especially for individuals who have attained complete remission through an AML-induction protocol^15^. Studies have suggested that HSCT can result in long-term survival and improved outcomes compared to conventional therapies^1^. However, some variations in outcomes have been observed, with one study indicating no significant difference in prognosis with HSCT, potentially influenced by a small patient cohort^10^. Comprehensive reviews addressing MS management have provided valuable insights into its multifaceted therapeutic approaches.

Gastric primary de-novo myeloid sarcoma is an uncommon and highly aggressive neoplasm. Enhancing our understanding of this condition is crucial for guiding treatment decisions and encouraging research efforts tailored to comprehending its unique characteristics within our patient population.

## Conclusion

In summary, gastric myeloid sarcoma presents as an extremely rare solid tumour, particularly when it arises without concurrent AML, thus adding complexity to its diagnosis. This case is the first of its kind globally, where gastric myeloid sarcoma presenting with pseudoachalasia. Documenting this unprecedented clinical presentation is imperative to broaden the understanding of the diverse spectrum of manifestations associated with gastric myeloid sarcoma.

## Ethical approval for case report

Medical records and histopathological slides of the case were retrospectively reviewed. The patient and family consent were obtained by the patient herself and patient’s family.

## Consent

Written informed consent was obtained from the patient for publication of this case report and accompanying images. A copy of the written consent is available for review by the Editor-in-Chief of this journal on request.

## Source of funding

None.

## Author contribution

N.E.M.A.J.: supervision. H.S.: writing—original draft, review and editing. M.A.N.: data curation. A.A.A.A.:resources. S.I.B.: methodology. R.S.R.: writing—original draft, review and editing. M.J.: writing—original draft, review and editing. Q.N.A.: writing—original draft, review and editing.

## Conflicts or interest disclosure

None.

## Research registration unique identifying number (UIN)

None.

## Guarantor

Husein Sarahneh.

## Availability of data and materials

The datasets used in this report are available from the corresponding author on reasonable request.

## Provenance and peer review

Not commissioned, externally peer-reviewed.
